# Emotional and cognitive correlates of Migraine: Links with headache burden and depression

**DOI:** 10.1192/j.eurpsy.2026.11044

**Published:** 2026-07-31

**Authors:** O. Samara Chatzianastasiou, A. Papadopoulou, M. Vikelis, E. Vousoura, P.-D. Stavrou, V. Efstathiou

**Affiliations:** 1 Department of Psychology, National and Kapodistrian University of Athens; 2 Second Department of Psychiatry, “Attikon” University General Hospital, National and Kapodistrian University of Athens; 3 Glyfada Headache Center, Athens, Greece

## Abstract

**Introduction:**

Migraine is a prevalent neurological disorder characterized by recurrent headaches and substantial impairment in daily functioning. Beyond the physical symptoms, psychological factors such as anxiety, depression, rumination, and cognitive reflection may shape the subjective burden of migraine.

**Objectives:**

This study aimed to compare individuals with and without migraine regarding headache burden and related psychological factors, and to identify demographic, clinical, and psychological correlates of depressive symptoms among individuals with migraine.

**Methods:**

The sample comprised 506 adults, including 325 with migraine and 181 controls. Participants completed the Hospital Anxiety and Depression Scale (HADS), the Rumination-Reflection Questionnaire (RRQ), the Migraine Disability Assessment Score (MIDAS), and a demographic and clinical questionnaire. Statistical analyses included group comparisons, correlational analyses, and multivariate regression models.

**Results:**

Individuals with migraine reported significantly higher depressive symptoms and greater headache-related functional burden compared to controls, whereas group differences in anxiety were not statistically significant. Gender effects emerged in cognitive reflection, with women scoring higher than men overall; however, an interaction indicated that women with migraine reported lower cognitive reflection than women without migraine. Within the migraine group, frequency of attacks correlated positively with anxiety, depression, rumination, and headache burden. Anger directed toward headaches was associated with elevated psychological distress, while longer illness duration was linked to lower cognitive reflection. Multivariate analyses identified headache burden, anxiety, rumination, and cognitive reflection as independent explanatory factors of depressive symptoms in migraine patients.

**Conclusions:**

The findings underscore the multifaceted psychological burden associated with migraine and highlight the importance of addressing both clinical and psychological variables in patient care. Depression in migraine appears to be independently influenced by headache-related disability, maladaptive cognitive-emotional styles, and lower levels of reflective processing. These results highlight the need for holistic approaches to migraine management that integrate psychological assessment and intervention alongside medical treatment.

**Disclosure of Interest:**

None Declared

